# The Ballot for Women

**Published:** 1875-11

**Authors:** 


### THE BALLOT FOR WOMAN.

The American Woman's Suffrage Society held its seventh annual meeting in this city lately. The sessions were well attended, and very interesting speeches were made. The speakers expressed great satisfaction at the rapid advance in public sentiment which the last year had developed; and the idea prevailed that within a year or two the right and duty to vote would be extended to women. The arguments in favor of allowing women to vote are absolutely unanswerable, and can not long consist of sneers and lofty smiles. Woman is the peer of man in intelligence, and is his superior in all the finer instincts. If she votes, it will be for worthy candidates and for honest measures. She will refine politics and politicians, as unquestionably as she has refined and adorned all things which she has touched—even man himself. When she collectively asks for the ballot, let her have it, by all means; nay, let us insist upon her taking it without the asking. But we are not able to learn that any large proportion of women are anxious for the ballot, or that they would accept it if it were offered to them. This lukewarmness is rather depressing to the hearts of those who not only believe that to vote is their right, but their duty as well. Woman is all-powerful; and when she has decided that she can do a grand work for herself and her race by employing the ballot, it will be hers within the year.

---

Great wants proceed from great wealth, but they are undutiful children, for they sink wealth down to poverty.

